# Inhalant abuse of 1,1-difluoroethane (DFE) leading to heterotopic ossification: a case report

**DOI:** 10.1186/1754-9493-2-28

**Published:** 2008-10-30

**Authors:** Jill Little, Barbara Hileman, Bruce H Ziran

**Affiliations:** 1 St. Elizabeth Health Center, Youngstown, OH, USA

## Abstract

**Background:**

Heterotopic ossification (HO) is the formation of mature, lamellar bone within soft tissues other than the periosteum. There are three recognized etiologies of HO: traumatic, neurogenic, and genetic. Presently, there are no definitively documented causal factors of HO. The following factors are presumed to place a patient at higher risk: 60 years of age or older, male, previous HO, hypertrophic osteoarthritis, ankylosing spondylitis, diffuse idiopathic skeletal hyperostosis, prior hip surgery, and surgical risk factors.

**Case presentation:**

A 33-year-old male, involved in a motor vehicle crash, sustained an irreducible acetabulum fracture/dislocation, displaced proximal humerus fracture, and an impacted pilon fracture. During the time of injury, he was intoxicated from inhaling the aerosol propellant used in "dust spray" cans (1,1-difluoroethane, C_2_H_4_F_2_). Radiographs identified rapid pathologic bone formation about the proximal humeral metaphysis, proximal femur, elbow, and soft tissue several months following the initial injury.

**Discussion:**

The patient did not have any genetic disorders that could have attributed to the bone formation but had some risk factors (male, fracture with dislocation). Surgically, the recommended precautions were followed to decrease the chance of HO. Although the patient did not have neurogenic injuries, the difluoroethane in dusting spray can cause damage to the central nervous system. Signals may have been mixed causing the patient's body to produce bone instead of tissue to strengthen the injured area.

**Conclusion:**

What is unusual in this case is the rate at which the pathological bone formation appeared, which was long outside the 4–6 week window in which HO starts to appear. The authors are not certain as to the cause of this rapid formation but suspect that the patient's continued abuse of inhaled aerosol propellants may be the culprit.

## Background

First described in 1883 by German physician Riedel, heterotopic ossification (HO) is defined as the formation of mature, lamellar bone within soft tissues other than the periosteum [1-3]. There are three recognized etiologies of HO: (1) traumatic – following fractures, dislocations, or operative procedures, (2) neurogenic – occurring after closed head injuries, insults to the spinal cord, or central nervous system infections, and (3) genetic (i.e. myositis ossificans progressiva – a rare, autosomal dominant disease) [1,2]. Although there are no definitively documented causal factors, HO is presumably the result of "the inappropriate differentiation of pluripotential mesenchymal cells into osteoblastic stem cells" [2]. Individuals over the age of 60, males, patients with previous HO bone, hypertrophic osteoarthritis, ankylosing spondylitis, diffuse idiopathic skeletal hyperostosis (DISH), prior hip surgery, and surgical risk factors place the patient at a higher risk for the formation of HO [1,2].

In the present case, we identify rapid pathogenic bone formation at multiple sites in a patient without any other significant risk factors except the abuse of an inhaled substance. Misuse or abuse of such compressed gases can result in difficulty breathing, alteration of the heart's electrical activity (irregular pulse, palpitations, inadequate circulation), abnormal kidney function, central nervous system depression, and death [4-8]. Neither literature nor material safety data sheets propose any skeletal risks related to overexposure or intentional abuse of 1,1-difluoroethane (DFE). To the knowledge of the authors, there is no existing literature suggesting the abuse of halogenated aliphatic aerosol propellants as a contributing factor to pathologic bone formation.

## Case presentation

**Figure 1:**
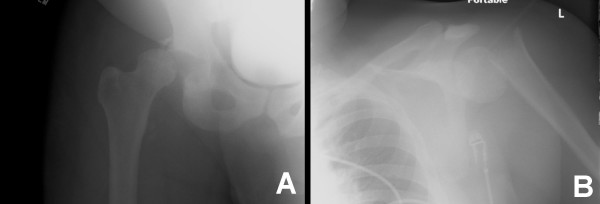
Radiographs of initial acetabular fracture dislocation and initial proximal humerus fracture.

**Figure 2:**
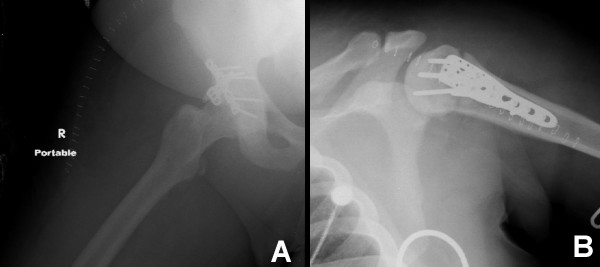
Post fixation radiographs of acetabular fracture dislocation and proximal humerus fracture.

A 33-year-old white male was admitted to the emergency department of a level I trauma center following a single-car motor vehicle crash. He presented with hemodynamic instability and a brief loss of consciousness prior to the accident. He was confused, disoriented to time and place, but oriented to person. The patient was a robust young man with no prior skeletal injuries but a known history of bipolar disorder and substance abuse. At the time of the injury, he had been inhaling the aerosol propellant used in "dust spray" cans (1,1-difluoroethane, C_2_H_4_F_2_) and was still noticeably intoxicated in the emergency department. Trauma evaluation revealed an irreducible fracture/dislocation of the right acetabulum, a displaced left proximal humerus fracture, and an impacted pilon fracture (Figure 1). The patient underwent open reduction and internal fixation (ORIF) for the acetabulum and humerus fractures and closed treatment for the distal tibial fracture on the same day (Figure 2). Deep vein thrombosis (DVT) prophylaxis was started, which consisted of mechanical and chemical prophylaxes. There was no evidence of any closed head injury.

**Figure 3:**
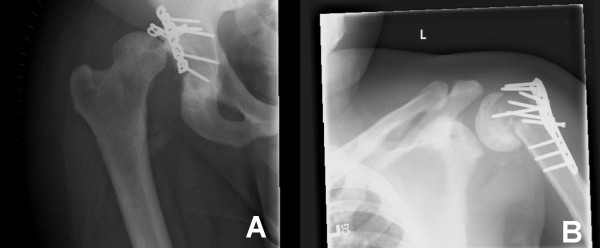
Radiographs of failed hardware after non-compliance with weight bearing, hip position, and ongoing substance abuse.

The patient failed to return for routine follow-up visits but presented to the emergency department one month later, after having tripped and fallen during one of his bouts of inhalant abuse. He waited several days after his fall before coming to the hospital and admitted to being noncompliant (premature weightbearing) with both the hip and shoulder. Radiographs demonstrated a dislocated hip as well as loss of fixation of the proximal humerus (Figure 3). The humeral hardware had failed and bone stock did not allow for suitable reconstruction. The patient was offered a resection arthroplasty for the hip but insisted on just a closed reduction and bracing of the hip. However, he was agreeable to surgical treatment of the humerus, which consisted of hardware removal. Because of his social living conditions, there was suspicion of potential infection as the cause for failure. As a result, routine intraoperative cultures were taken. There was no intraoperative evidence of infection, but cultures demonstrated one colony-forming unit of coagulase negative *Staphylococcus*. Appropriate IV antibiotics were administered with no further clinical symptomatology. During further follow-up visits, he continued with his substance abuse and noncompliance according to his family. At these follow-up visits, there was progressive luxation of his hip with the development of a Hill-Sachs type lesion of the femoral head. In addition, the humeral head appeared avascular with collapse of the humeral head along with erosive changes. At this point, the patient complained of ongoing pain that he claims was partly responsible for his substance abuse problems (pain control). With his history of noncompliance, on-going substance abuse as well as an unmanaged psychotic disorder, he was not considered a good candidate for any type of reconstructive surgery. He was again offered resection arthroplasty and this time agreed. The surgery, however, was postponed and rescheduled as the patient insisted on getting more inhalants on his way to the hospital.

**Figure 4:**
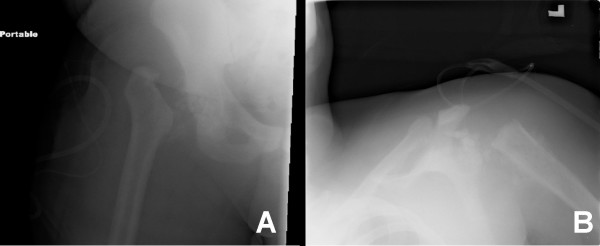
Radiographs of resection arthroplasties of hip and shoulder.

**Figure 5:**
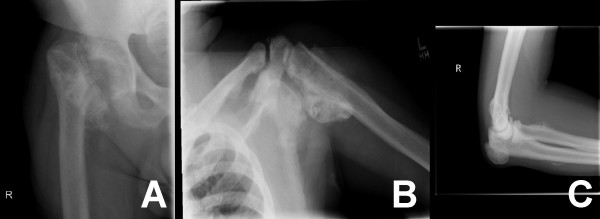
Radiographs of hetertopic bone formation and metaphyseal bone and appearance of sessile exostosis.

Girdlestone resection arthroplasty of the right hip and left humerus were performed for pain relief (Figure 4). At this juncture, multiple cultures of both operative sites were negative. Weightbearing was allowed as tolerated with bracing for both the acetabulum and humerus. The pilon fracture went on to heal with a mild dorsiflexion deformity and was not causing him any problems. Four months after injury, there was no evidence of any pathologic bone formation. During the course of the next several months, the patient's abuse of the halogenated aliphatic aerosol propellant increased to the point that it interfered with his rehabilitation and he remained at home except for efforts to procure more inhalant. Physical therapy discontinued treatment, because he was reported to be using the inhalants during his sessions. The patient did not return for a post-surgical follow-up visit until three months later. At that time, he had limited motion of the hip and shoulder without having a significant amount of discomfort. He also described subcutaneous "bumps" and swellings around the arm and elbow. Radiographs identified pathologic bone formation about the proximal humeral metaphysis, proximal femur, and the elbow. The appearance of the bone was opaque with well-rounded edges and a base that appeared attached to the cortex. There was also pathologic bone formation in the soft tissues. In this context, we felt that he not only had heterotopic bone but also had what was reminiscent of an exostotic process (Figure 5). Additional x-rays, a CAT scan, and bone biopsy were ordered for the patient, but he has refused to comply with his physicians' instructions to return for further follow-up care.

## Discussion

To rule out any signs or factors that may have contributed to HO in the patient, we reviewed pertinent medical, social and surgical history. However, the patient does not have a history of any of the contributing genetic disorders (fibrodysplasia ossificans progressiva, progressive osseous heteroplasia, or Albright's hereditary osteodystrophy). While HO may also be a result of the trauma, we do not believe that this is the case here. The patient did undergo ORIF for a displaced fracture dislocation of the acetabulum and displaced proximal humerus. Subsequent surgeries included hardware removal and resection arthroplasties in both areas. However, all recommended precautions to reduce the risk of HO were taken: atraumatic surgical techniques, careful excision, thorough irrigation and debridement, and administration of antibiotic prophylaxis. Additionally, the pathologic bone formation was not evident in any radiographs until 6 months following the initial injuries and 3 months after his resection surgery. This is long outside the 4–6 week window in which HO starts to appear on plain radiographs [3].

The final recognized cause of HO formation is neurogenic with either an insult to the central nervous system (CNS) including spinal trauma and head injuries or neurologic conditions such as encephalitis, meningitis, myelitis, tetanus, brain tumors, epidural abscess, or subarachnoid hemorrhage [1]. Campos et al. hypothesize that the formation of HO in neurogenic injuries may be related to proprioception dysfunction. Perhaps because of "mixed signals," stimuli that were intended to strengthen tissue resistance resulted in HO [9].

There was minor evidence of this patient sustaining any neurogenic injuries as a result of the motor vehicle accident. The patient's Glascow Coma Score (GCS) was a 15 upon arrival to the emergency department, and his head was found to be normal with no swelling, ecchymosis, tenderness, lesions, abrasions, or lacerations. However, one of the hazardous health effects related to the inhalation of high concentrations of halogenated aliphatic aerosol propellants is damage to the CNS [4-8]. Furthermore, the most commonly found chemical ingredient in dusting sprays is DFE. Some of the side effects of DFE were apparent when the patient displayed metabolic abnormalities and high blood pressure during his final surgery, which could not be attributed to anything other than his increased abuse of DFE.

While the patient had some risk factors (male, fracture with dislocation) [1,2], the authors feel that the biggest influence may have been the exorbitant inhalation of DFE. This substance may have exacerbated or accelerated any predisposition the patient had to developing HO.

## Conclusion

HO following trauma and surgical intervention is not uncommon [1-3]. What is unusual in this case is the rate at which the pathological bone formation appeared, which was three months after the Girdlestone resection arthroplasties. In addition, radiographs obtained when the patient first presented did not reveal any HO at the time. The authors are not certain as to the cause of this rapid formation but suspect that the patient's continued abuse of inhaled aerosol propellants may be the culprit. Interestingly, the HO is not limited to the areas where the resection arthroplasties were performed (although they are most abundant here), but it is present throughout the body.

## Consent

Written informed consent was obtained by the patient for publication of this case report.

## Competing interests

The authors declare that they have no competing interests.

## Authors' contributions

BZ conceived of the study, participated in its design, and helped draft the manuscript. JL participated in the study design and data analysis plan and drafted and revised the manuscript. BH participated in the study design and helped revise the manuscript. All authors have read and approved the final manuscript.

## References

[B1] Balboni TA, Gobezie R, Mamon HJ (2006). Heterotopic ossification: Pathophysiology, clinical features, and the role of radiotherapy for prophylaxis. Int J Radiat Oncol Biol Phys.

[B2] Naraghi FF, DeCoster TA, Monein MS, Miller RA, Rivero D (1996). Heterotopic ossification. Orthopedics.

[B3] Chao ST, Joyce MJ, Suh JH (2007). Treatment of heterotopic ossification. Orthopedics.

[B4] What is Inhalant Abuse > Dangers. http://www.inhalant.org/inhalant/dangers.php.

[B5] Material Safety Data Sheet "Dymel" 152a. http://www.sisweb.com/referenc/msds/dustoff.pdf.

[B6] Matehson Tri Gas Material Safety Data Sheet. http://www.matheson-trigas.com/pdfs/msds/MAT26280.pdf.

[B7] Material Safety Data Sheet. http://www.blowoff.com/msds/152amsds.html.

[B8] Material Safety Data Sheet. http://www.tulstar.com/documents/HFC-152A.pdf.

[B9] Campos de Paz A, Carod Artal FJ, Kalil RK (2007). The function of proprioceptors in bone organization: A possible explanation for neurogenic heterotopic ossification in patients with neurological damage. Med Hypotheses.

